# Editorial: New trends in regional analgesia and anesthesia

**DOI:** 10.3389/fmed.2023.1256371

**Published:** 2023-08-01

**Authors:** Shun-Ming Chan, Po-Kai Wang, Jui-An Lin

**Affiliations:** ^1^Department of Anesthesiology, Tri-Service General Hospital, National Defense Medical Center, Taipei City, Taiwan; ^2^Department of Anesthesiology, Hualien Tzu Chi Hospital, Buddhist Tzu Chi Medical Foundation, Hualien, Taiwan; ^3^School of Medicine, Tzu Chi University, Hualien, Taiwan; ^4^Department of Anesthesiology, School of Medicine, Chung Shan Medical University, Taichung, Taiwan; ^5^Department of Anesthesiology, Chung Shan Medical University Hospital, Taichung, Taiwan; ^6^Center for Regional Anesthesia and Pain Medicine, Chung Shan Medical University Hospital, Taichung, Taiwan; ^7^Department of Anesthesiology, School of Medicine, College of Medicine, Taipei Medical University, Taipei City, Taiwan; ^8^Department of Anesthesiology, School of Medicine, National Defense Medical Center, Taipei City, Taiwan; ^9^Center for Regional Anesthesia and Pain Medicine, Wanfang Hospital, Taipei Medical University, Taipei City, Taiwan

**Keywords:** analgesia, anesthesia: conduction, artificial intelligence, anesthesia, analgesia: multimodal

Two review articles examined six peripheral nerve block techniques after arthroscopic shoulder surgery in terms of efficacy and adverse effects (Liu et al.; Jiangping et al.). Liu et al. presented the first network meta-analysis of postoperative pain regimens after arthroscopic shoulder surgery has been conducted. In comparison to other peripheral nerve blocks, interscalene brachial plexus blocks reduced pain and opioid consumption better, but had a higher rate of adverse events. There is a risk of diaphragmatic paresis due to the location of the interscalene insertion near the phrenic nerve. Jiangping et al. also proposed the same idea. However, Hussain et al. indicated that suprascapular block and interscalene block don't differ clinically in analgesia and suprascapular block has fewer complications (1). In the future, high–quality randomized controlled trials should continue to examine the best multimodal analgesic regimen for perioperative pain after shoulder arthroscopy. In another systemic review article (Fenta et al.), post-spinal anesthetic shivering was evaluated based on injection of local anesthetics into subarachnoid spaces. Fenta et al. found that patients receiving intravenous ketamine had fewer instances of nausea, vomiting, and bradycardia compared with patients receiving intravenous tramadol. Ketamine is a competitive N-methyl-D-aspartate receptor antagonist that plays a major role in inhibiting postoperative shivering, and it is thought that its anti-shivering effect may be through the action on the hypothalamus or through the β-adrenergic effect of norepinephrine. Generally speaking, postoperative shivering is a frequent complication of anesthesia. Shivering is believed to increase oxygen consumption and the risk of hypoxemia, as well as induce lactic acidosis and catecholamine release. Prevention and management of shivering are critical as it may reduce the potential for many adverse effects. However, the precise mechanism by which these medications stop shivering is not well known.

This Research Topic also includes an original study on the impact of pregabalin on the minimum alveolar concentration (MAC) of inhaled anesthetics. Pregabalin is effective as preemptive analgesia for neuropathic pain. Over the past several years, it has increasingly been used perioperatively to reduce postoperative pain intensity, and opioid use, and prevent post-operative pain. Pregabalin is still included in many multimodal perioperative analgesic regimens. Müller et al. presented that administration of 300 mg pregabalin preoperatively lowers the MAC of sevoflurane by 33%, while administration of 150 mg pregabalin did not result in a significant reduction in MAC. Pregabalin, depending on the dose, had a slight decrease in postoperative pain levels, but it also had an increase in side effects, such as nausea and vomiting, dizziness, and headache.The results are consistent with these previous studies indicating that pregabalin spares inhalation anesthetic, maintains hemodynamics, and optimizes postoperative analgesia (2). Obtaining more quality evidence in this field is crucial, as only a few studies exist in this area.

Over the past few years, the opioid epidemic has emerged as one of the world's most critical challenges. Multimodal analgesia (MMA) also falls under this Research Topic. Previous studies have shown that the combination with multimodal analgesia, enhances recovery after surgical procedure, reduces perioperative use of opioids, and later on, their adverse effects (3). Conversely, the Research Topic reported opposing results to the previous study, where the use of the pectoralis nerve block has not significantly reduced the use of perioperative opioids relative to MMA alone in elective breast surgery (Uribe et al.). Most of prospective and retrospective studies, systematic reviews, and meta-analyses published in recent years demonstrated that the combined pectoral nerve block during anesthesia reduces the severity of postoperative pain and the total amount of perioperative opioid. However, following possible analysis, this can be attributed to the intrinsic limitation of a retrospective study, the small sample size and the inability to collect data on opioid use over 24 h. Additionally, dose and regimen of MMA were not consistent across these studies. The use of gabapentinoids in postoperative pain management schemes has linked to a high incidence of adverse events such as conscious disturbances and vertigo that hinder early mobilization and delay recovery.

This Research Subject also comprises one scoping review regarding artificial intelligence (AI) in ultrasound-guided regional anesthesia (Viderman et al.). The theme of AI has become very hot recently because of Nvidia founder Jensen Huang. Viderman et al. indicated that AI solutions could be helpful in identifying anatomical cues, reducing or even avoiding possible complications. AI solutions can assist in identifying anatomical markers and reduce or even prevent potential complications. As a result, strong collaboration between clinicians and engineers is critical. Attracting medical students and talented practitioners to the anesthesia profession will take a multipronged approach and time. Perhaps AI can solve this problem more quickly. In the past, many physicians (except for anesthesiologists with expertise in regional anesthesia) regarded regional anesthesia as too complex and intimidating. Another barrier may be the longer time to perform regional anesthesia than conventional pain management. AI-based devices may potentially facilitate the acquisition and interpretation of ultrasound-guided regional anesthesia images. Such technology could improve the performance of ultrasound for regional anesthesia by non-experts, which could expand patient access to these techniques. More research is required to demonstrate the effectiveness of AI in supporting training and clinical practice.

## Author contributions

S-MC prepared the draft. P-KW and J-AL revised the manuscript. All authors contributed to the article and approved the submitted version.
